# Karst-bauxite formation during the Great Oxidation Event indicated by dating of authigenic rutile and its thorium content

**DOI:** 10.1038/s41598-023-35574-x

**Published:** 2023-05-27

**Authors:** Alexandre Raphael Cabral, Armin Zeh

**Affiliations:** 1 Centro de Pesquisas Professor Manoel Teixeira da Costa (CPMTC), Instituto de Geociências, Universidade Federal de Minas Gerais (UFMG), Belo Horizonte, Brazil; 2 Centro de Desenvolvimento da Tecnologia Nuclear (CDTN), Belo Horizonte, Brazil; 3 Karlsruher Institut für Technologie (KIT), Campus Süd, Institut für Angewandte Geowissenschaften, Mineralogie und Petrologie, Karlsruhe, Germany

**Keywords:** Mineralogy, Geochemistry, Economic geology, Precambrian geology

## Abstract

Aluminium (Al)-rich palaeosols—i.e., palaeobauxite deposits—should have formed in karst depressions in carbonate sequences as a result of acidic solutions from oxidative weathering of sulfide minerals during the Great Oxidation Event (GOE), but no GOE-related karst-palaeobauxite deposits have so far been recorded. Here, we report results of in situ uranium–lead (U–Pb) dating of detrital zircon and spatially associated rutile from a metamorphosed Al-rich rock within a dolomite sequence in the Quadrilátero Ferrífero (QF) of Minas Gerais, Brazil, known as the Gandarela Formation. Rutile grains are highly enriched in thorium (Th = 3–46 ppm; Th/U ratio = 0.3–3.7) and yielded an isochron, lower-intercept age of ca. 2.12 Ga, which coincides with the final phase of the GOE—i.e., the Lomagundi event. The rutile age represents either authigenic growth of TiO_2_ enriched in Th, U and Pb during bauxite formation, or subsequent rutile crystallisation during metamorphic overprint. Both cases require an authigenic origin for the rutile. Its high Th contents can be used as a palaeoenvironmental indicator for decreased soil pH during the GOE. Our results also have implications for iron (Fe)-ore genesis in the QF. This study demonstrates that in situ U–Th–Pb-isotope analyses of rutile can place tight constraints on the age and nature of palaeosols.

## Introduction

Rutile is a common accessory mineral of detrital origin in sedimentary and metasedimentary rocks. Detrital rutile has characteristically low contents of Th, generally less than 0.5 ppm Th^1^. The detrital nature of rutile reflects the very low solubility of rutile in H_2_O^2^, which in turn explains much of the Ti content as detrital rutile in residual products of deep weathering, such as bauxite deposits. Titanium has been demonstrated to be immobile in bauxite deposits, despite the formation of authigenic TiO_2_ as anatase during bauxitisation^3^.

Bauxitisation requires the removal of Fe from soils as Fe^+2^, at low Eh conditions that are compatible with pre-2.4-Ga levels of atmospheric oxygen, before the GOE^4^, recently redefined as the ‘Great Oxidation Episode’^5^. While bauxitisation is possible under non-oxidative weathering, some Archaean examples of which have been recorded as metamorphosed rocks rich in Al^6^, the formation of authigenic anatase in bauxitic profiles implies oxidative weathering of ilmenite (FeTiO_3_), a major detrital source of Ti in present-day soils^7^. Authigenic anatase, as observed in present-day soils, should also have existed in palaeosols, where it is expected to be subsequently converted to rutile during prograde metamorphism^8^. Authigenic rutile has nonetheless gone unrecognised in metamorphosed bauxitic palaeosols.

Bauxitic palaeosols formed in karst depressions have not been documented during the GOE^6^. The apparent absence of karst bauxite is at odds with the GOE oxidative weathering, whereby the oxidation of sulfide minerals would have generated acidic terrestrial waters^9^. The latter would have produced karst depressions in carbonate sequences, where karst bauxites could have developed with relative Ti enrichment due to the formation of authigenic TiO_2_. Here, we describe for the first time the occurrence of authigenic TiO_2_ as rutile with high contents of Th in a metamorphosed karst bauxite from the Gandarela Formation of the QF. We demonstrate that authigenic rutile can be recognised by conventional reflected-light microscopy, and used as a timepiece of oxidative weathering during the GOE by applying in situ LA–ICP–SF–MS (laser ablation–inductively coupled plasma–sector field–mass spectrometry) for U–Th–Pb isotopes. We further indicate implications for the Fe-ore genesis in the QF of Minas Gerais, a world-class Fe-ore district in Brazil.

## Study area and geological setting

**Figure 1:**
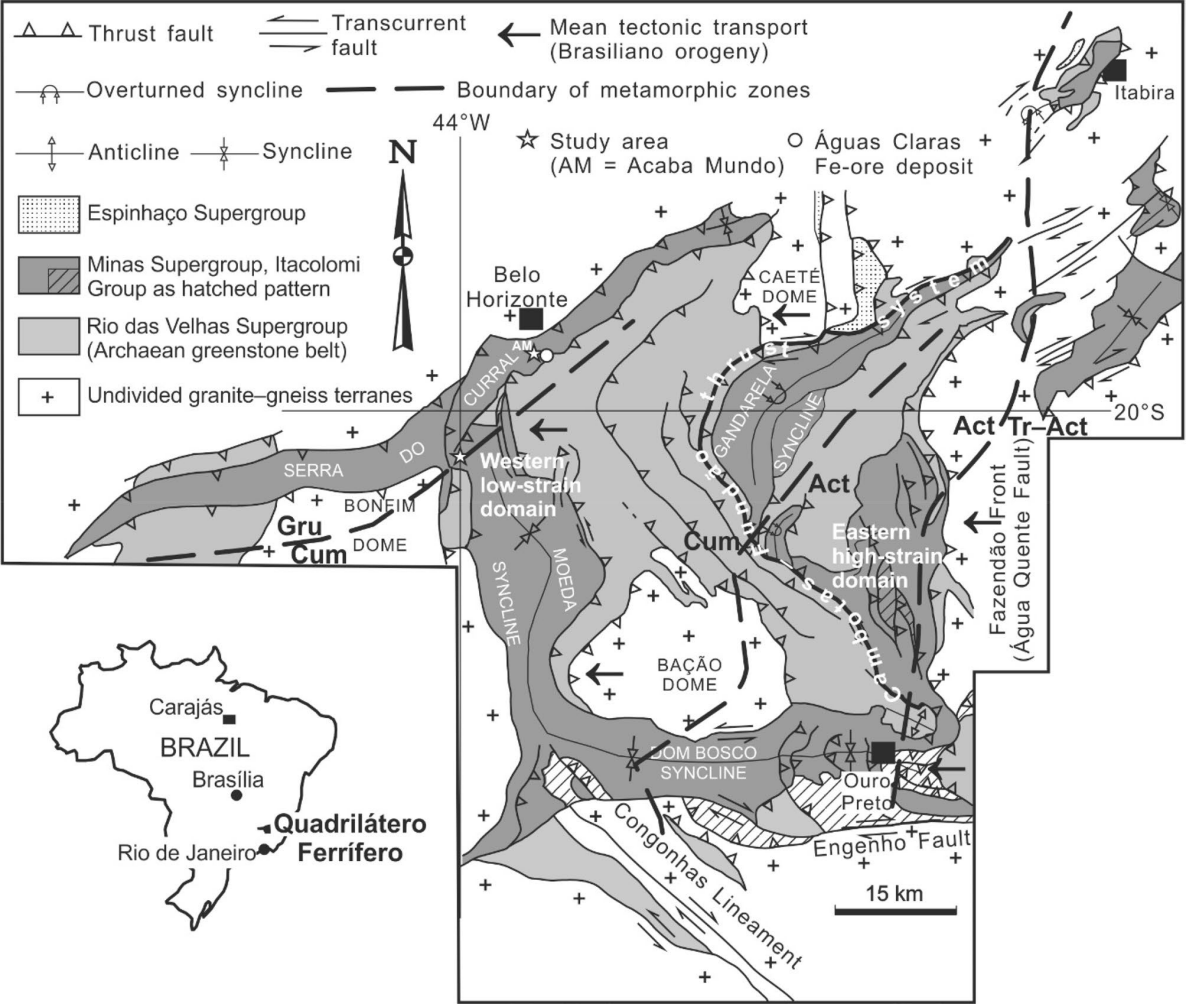
Location of the study area (Acaba Mundo) in the geological context of the Quadrilátero Ferrífero of Minas Gerais. The map is adapted from that presented in Rosière et al.^33^, following the work of Dorr^10^ and Harder and Chamberlin^12^. Metamorphic zones, based on the distribution of amphibole minerals in itabirite^42^, are as follows: *Gru* grunerite, *Cum* cummingtonite, *Act* actinolite, *Tr–Act* tremolite–actinolite.

The study area is a decommissioned dolomite quarry on the outskirts of Belo Horizonte (Fig. 1). The quarry, known as Acaba Mundo, exposes dolomitic rocks of the Gandarela Formation, below which is an itabirite sequence, the Cauê Itabirite of Dorr^10^, likely deposited at 2.65 Ga^11^. Itabirite is a metamorphosed rock of alternating bands rich in either hematite or magnetite, and bands rich in gangue minerals, mostly quartz. The Cauê Itabirite and the Gandarela Formation, which lies with gradational contact on the former, comprise the Itabira Group of Dorr^10^, or the Itabira iron formation of Harder and Chamberlin^12^. The Cauê Itabirite forms ridges that host Fe-ore deposits in the QF. One of such ridges is Serra do Curral, on the southern flank of which is Águas Claras, an itabirite-hosted world-class Fe-ore deposit^13^. In its vicinity, on the northern flank of the ridge, is the Acaba Mundo dolomite quarry.

The quarry dolomite rocks are delimited to the north by predominantly clastic rocks of the Piracicaba Group, which disconformably overlies the Itabira Group. The disconformable contact between the two groups is an erosional surface^10^, the age of which has been constrained at 2141 ± 6 Ma by U–Pb dating of zircon grains from a deeply weathered sequence of pillow lavas^14^. This Palaeoproterozoic age is interpreted as the subaqueous volcanism that covered the erosional unconformity between the Itabira and the Piracicaba groups. The subaqueous volcanism occurred either shortly before or at the onset of the final phase of the Transamazonian orogeny, marked by gneiss-dome emplacement at 2095 ± 65 Ma^15^. Another orogenic event, the Brasiliano orogeny, was superimposed between 0.62 and 0.50 Ga, this time span being determined in the south-eastern QF^16^.

## Sample material

The sample material is a dolomite-hosted body of non-foliated metamorphic rock. It has cm-long laths of a kyanite-like mineral. The mineral is extensively altered to a soft, fine-grained mineral assemblage in shades of light green to grey. The rock was sampled from the dump of an exploratory adit that was driven into the quarry dolomite. The excavated material from the adit was too aluminous for dolomite mining.

## Results

**Figure 2:**
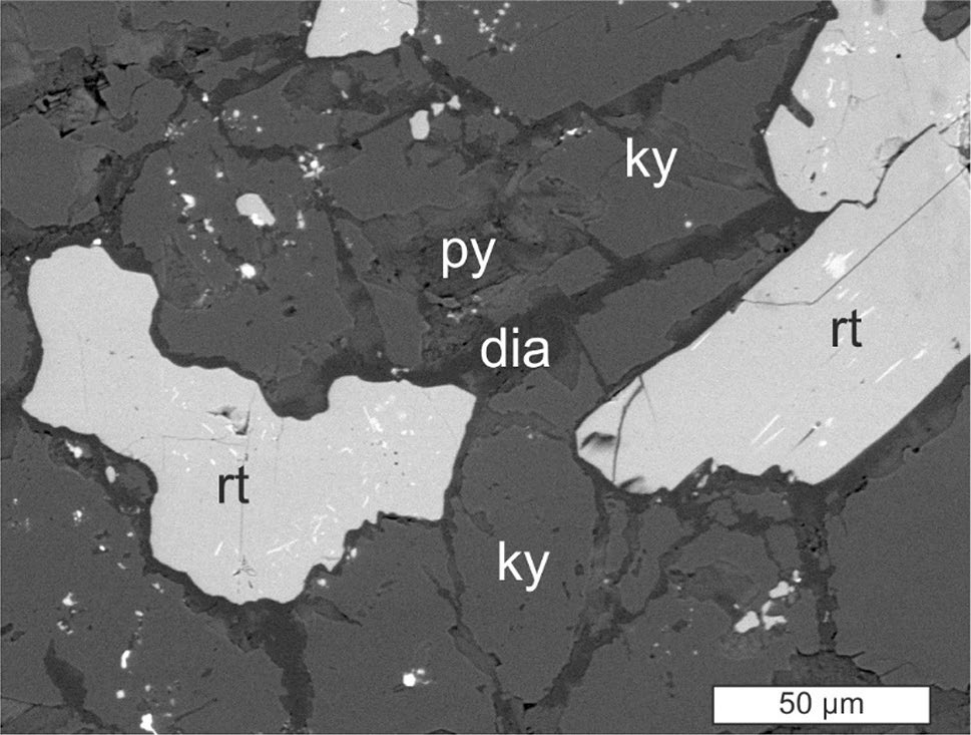
Backscattered-electron (BSE) image of an Al-rich rock. Pyrophyllite (py) and diaspore (dia) occur as overprint on kyanite (ky), with which rutile (rt) is spatially associated. Some rutile grains have lamellae and patches of a C﻿r–Fe-oxide mineral (white).

**Figure 3:**
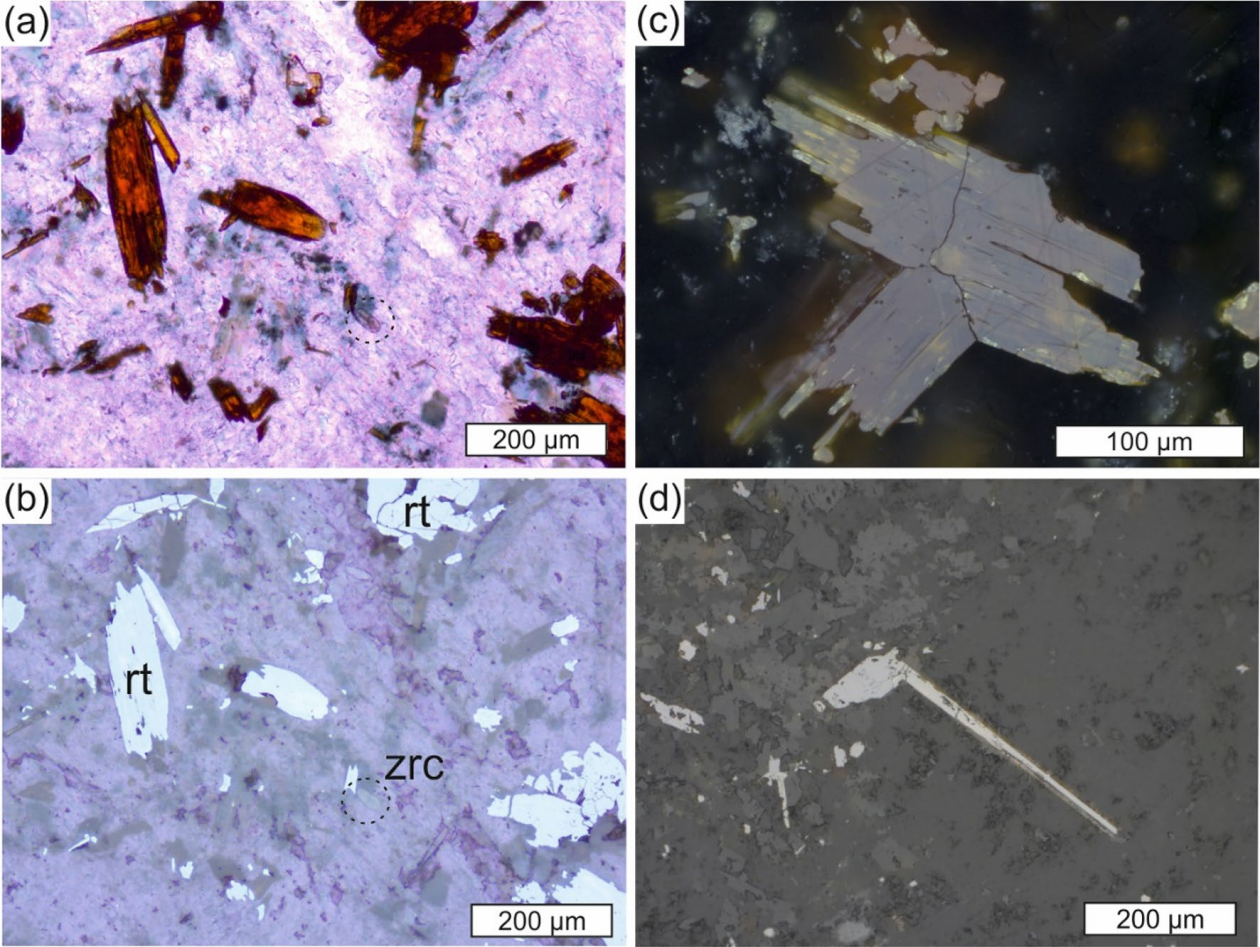
Photomicrographs of rutile in a kyanite–muscovite–diaspore–pyrophyllite rock. (**a**) Rutile grains show brownish-red colour in plane-parallel transmitted light. (**b**) The same rutile (rt) grains depicted in a appear grey in reflected light (air). Dashed circles in a and b denote a zircon (zrc) grain. (**c**) Twinned rutile (centre, reflected light, oil immersion). (**d**) Rutile and its protrusion stemming from a twinned contact (centre, reflected light, air).

**Figure 4:**
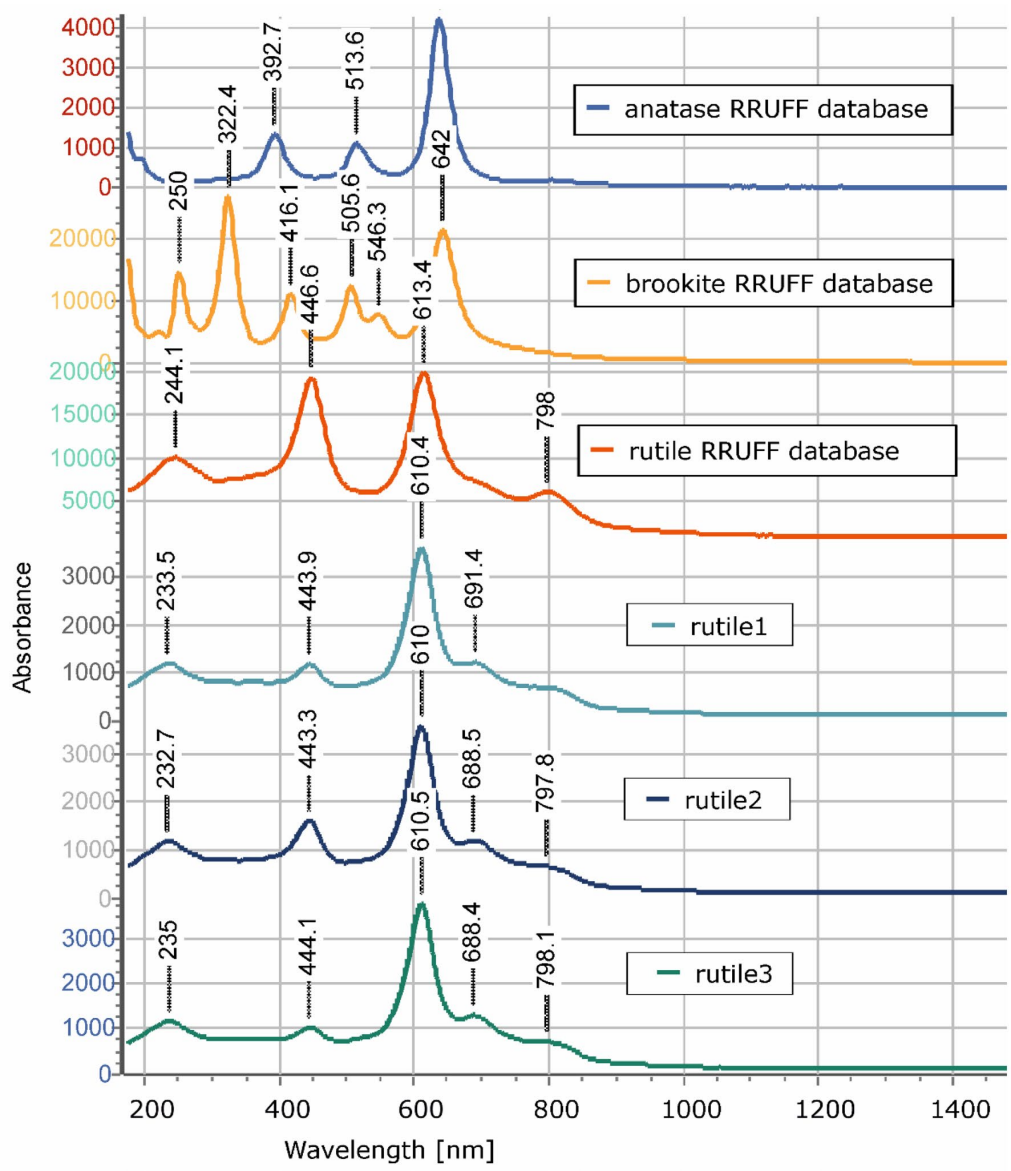
Raman spectra of three rutile grains, rutile 1, 2 and 3, which occur in the Al-rich rock of Figs. 2 and 3, in comparison with Raman spectra of TiO_2_ polymorphs—i.e., anatase, brookite and rutile.

The aluminous rock within the dolomite sequence of the Gandarela Formation has pyrophyllite, diaspore, muscovite and kyanite as the main mineral components (Supplementary Information Fig. S1 and Table S1). Pyrophyllite and diaspore occur as overprints on kyanite (Fig. 2). Rutile, a widespread accessory mineral in the rock (Fig. 3a,b), exhibits its distinctive internal reflections, abundant and very bright^17^, as well as its characteristic geniculated twin (Fig. 3c). This elbow twin may form elongated protrusions, reaching about 0.5 mm in length (Fig. 3d). Raman spectra confirmed the reflected-light identification of rutile (Fig. 4). Another omnipresent accessory mineral is zircon (Fig. 3a,b), which is typically euhedral to round and shows no outgrowths.

**Figure 5:**
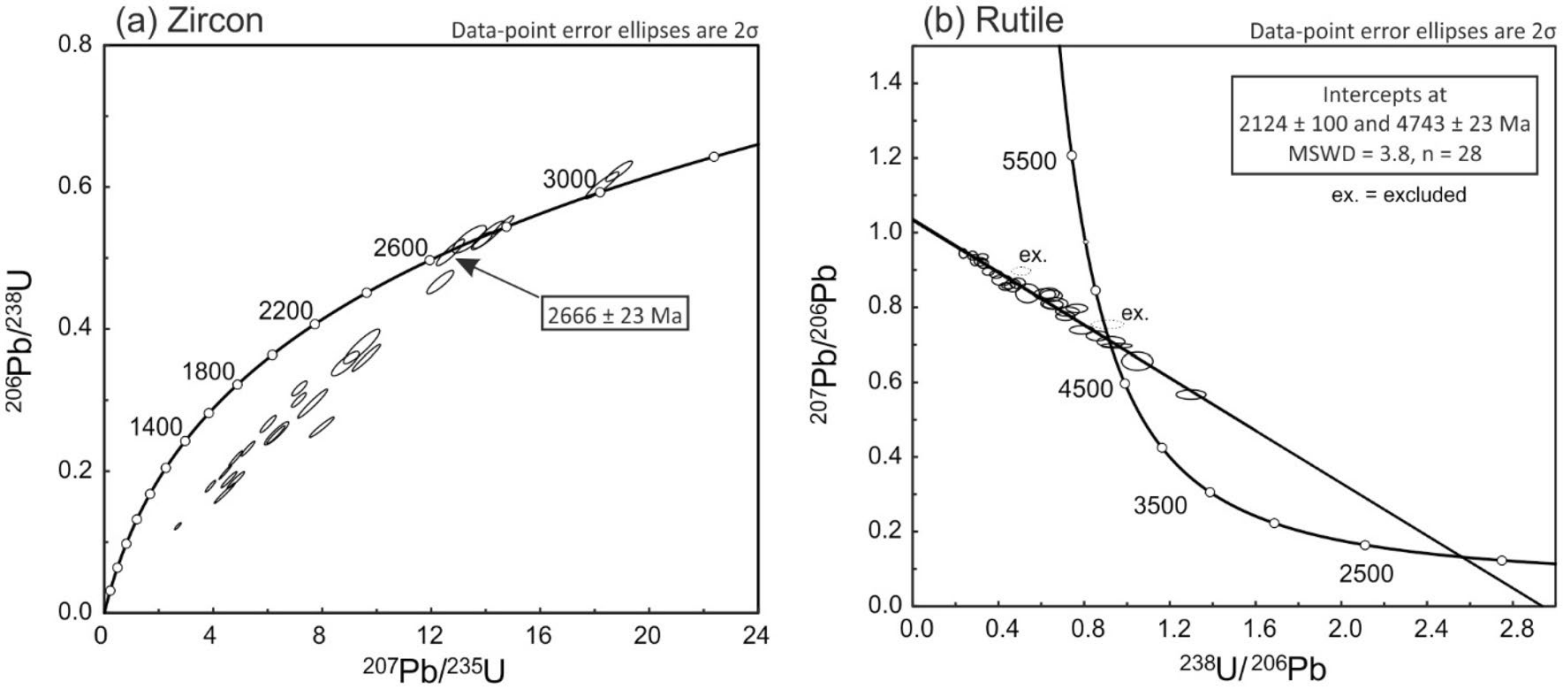
Concordia and Tera-Wasserburg plots for zircon (**a**) and rutile (**b**), respectively.

The thin section investigated has a total of 20 zircon grains that could be measured by LA–ICP–SF–MS, some of them twice (Supplementary Information Table S2). Most analyses yielded discordant U–Pb ages, but seven grains returned concordant ages between ca. 3000 and ca. 2670 Ma (Fig. 5a). The youngest zircon has an age of 2666 ± 23 Ma. Measurements of 30 rutile grains gave variable ^238^U/^206^Pb ratios, with 28 analyses defining an isochrone with a lower-intercept ^206^Pb/^238^U age of 2124 ± 100 Ma (Fig. 5b; Supplementary Information Table S3). All grains show high Th contents (3–46 ppm) and elevated ratios of Th/U (0.33–3.75) and Pb/U (2–16).

## Discussion

### Protolith nature, detrital zircon versus authigenic rutile and the GOE

The unusually high whole-rock content of Al, mineralogically represented by kyanite that was altered to pyrophyllite, diaspore and muscovite, and the paucity of quartz and Fe-rich minerals indicate that Al was residually enriched in the protolith. Residual Al enrichment needs to be reconciled with Fe removal within the dolomite sequence of the Gandarela Formation. A likely reconciliation scenario is bauxitisation during karst weathering^3^. The karst-bauxite scenario is corroborated by the restricted occurrence of Al-rich minerals in pockets within the dolomite, and the detrital nature of zircon grains with a wide range of concordant U–Pb ages, between ca. 3000 and ca. 2660 Ma. These data imply that: (1) Archaean granite-gneiss basement rocks provided detritus to karst depressions; and (2) part of the Gandarela dolomite sequence was exposed to karst weathering and subsequent bauxitisation.

Bauxitisation under oxidative weathering is expected to form authigenic TiO_2_, which is a residual product of ilmenite oxidation and progressive Fe removal^7,18^. Coarsening of authigenic TiO_2_ as nanocrystalline anatase particles takes place at higher temperatures and involves twinning and rutile growth, as modelled from kinetic experiments^19^. Twinning and rutile growth during prograde metamorphism would then be expressed in the widespread occurrence of coarse-grained twinned rutile in the metamorphosed aluminous rock (Fig. 3), interpreted to have been a karst-bauxite deposit. The rutile age of 2124 ± 100 Ma reflects the timing of either authigenic TiO_2_ crystallisation during karst-bauxite formation, or its subsequent metamorphic overprint.

The authigenic nature of the rutile grains can further be examined considering their high Th contents (3–46 ppm), resulting in elevated Th/U ratios (up to 3.7), far higher than those obtained from detrital rutile grains supplied from magmatic or metamorphic rocks (mostly < 0.003)^20^. The very low Th content results from the much larger size of Th^4+^ compared to Ti^4+^, preventing its uptake during rutile growth. Nonetheless, diagenetically grown rutile in sandstone is reported to contain up to 35 ppm Th and high Th/U up to 8.75^21^. Such high Th contents and Th/U ratios can only be achieved by a mechanism of successive crystallisation of amorphous to poorly crystalline TiO_2_ or aggregates of nanocrystalline TiO_2_, leading to Th uptake into interstitial positions^22,23^, in line with Th immobility during oxidative weathering^3^. Enhanced Th uptake is known from karst-bauxite anatase^23^, and from authigenic rutile in low-grade sandstones of the Moeda Formation^22^, underlying the Itabira Group.

Authigenic grains of rutile from the Moeda Formation yielded concordant U–Pb ages between 2245 and 2110 Ma, interpreted to date the timing of fluid–rock interaction during the Transamazonian orogeny^22^. The ages overlap with the lower-intercept age of 2124 ± 100 Ma obtained from the authigenic rutile investigated here (thereafter referred to as the bauxite rutile), but are ca. 500 Myr younger than the age of the youngest detrital zircon (2666 ± 23 Ma) in the karst bauxite, defining the maximum depositional age. The bauxite-rutile age also overlaps with an age of 2141 ± 6 Ma for the subaqueous volcanism that covers the erosional unconformity between the Itabira and the Piracicaba groups^14^. Furthermore, it is within error identical to an age of 2095 ± 65 Ma suggested to date the thermal overprint during the Transamazonian gneiss-dome emplacement in the QF^15^. However, it is significantly older than an age of 499 ± 3 Ma estimated for metamorphic rutile in the Sítio Largo amphibolite^24^, recording the timing of the structural-metamorphic overprint during the Brasiliano orogeny.

In combination, stratigraphical relationships and geochronological data indicate that karstification and bauxitisation of karst-filling clastic sediments took place within the GOE, specifically at its final stage, the Lomagundi event (see below). The erosional unconformity between the Itabira and the Piracicaba groups, dated at 2.14 Ga^14^, constrains the minimum age for the uplift of the Gandarela dolomite rocks and their exposure to karst weathering, during the gneiss-dome emplacement in the QF^15^, immediately followed by metamorphism.

### Titanium and Th mobility during the GOE

The bauxite rutile of Fig. 3d has such a long twin branch that requires input of mobile Ti. At least local mobility of Ti has been recognised in soils and sediments^25,26^. In such environments, Ti can be mobilised in the presence of organic acids. A crucial point is that metal complexing by organic ligands is mediated by highly oxidised compounds that resemble humic substances^27^. The Ti input to form the elongated protrusion of Fig. 3d is consistent with a soil where pyrite oxidation led to decreased soil pH, which increased phosphate loss and produced relative Ti enrichment, as observed in palaeosols developed during the GOE, in particular during the Lomagundi event at ca. 2.22–2.06 Ga^28^. In this regard, the high contents of common Pb determined in the bauxite rutile (Supplementary Information Table S3) could have been derived from aqueous solutions containing Pb from oxidised sulfide minerals. Interestingly, dissolved Pb^+2^ promotes rutile aggregation at reduced pH^29^.

Importantly, reduced pH leads to increased solubilities of Th in such a manner that the concentration of soluble Th increases by seven orders of magnitude from pH 6 to pH 4 at temperatures between 18 and 25 °C^30^. Therefore, high contents of Th (> 1 ppm) in authigenic rutile might be an indicator of decreased soil pH, possibly involving organic acids, during the GOE. It should be noted, however, that Th solubilities also increase in alkaline CaCl_2_ solutions by six orders of magnitude from pH 10 to pH 12 at temperatures between 17 and 25 °C^31^.

### Bauxitisation and Fe-ore genesis

An upgrade in the Fe content of itabirite to form high-grade orebodies of massive, hard hematite has been attributed to the metamorphic overprint of the Transamazonian orogeny, an interpretation supported by a U–Pb age of 2034 ± 11 Ma for monazite that coexists with granoblastic hematite after magnetite^32^. The Fe upgrade involved leaching of gangue minerals by hydrothermal fluids^33^. Much of the leaching of gangue minerals could have occurred in itabirite domains exposed to weathering, concomitant with the ca. 2.14-Ga bauxitisation of terrigenous material that filled karst depressions in the overlying dolomite sequence of the Gandarela Formation, as indicated by the results of this study.

## Conclusions

Authigenic rutile from an Al-rich rock is characterised by coarse-grained twinned crystals that are considerably younger than spatially associated grains of detrital zircon. Recognising the authigenic nature of rutile in extremely aluminous rocks enables its use as a palaeoenvironmental timepiece because: (1) authigenic TiO_2_ is formed from the oxidative weathering of ilmenite, as recorded from modern weathering profiles; (2) high Th contents (> 1 ppm) attest to either acidic or alkaline soil conditions; (3) U–Pb dating of authigenic rutile and detrital zircon places age constraints. These criteria define the extremely Al-rich rock as metamorphosed karst bauxite, originally developed during the GOE over the uplifted part of the Gandarela dolomite sequence. High Th contents in authigenic rutile are consistent with decreased soil pH during the GOE.

## Methods

One sample of the aluminous rock was cut for polished-thin-section preparation; an aliquot of it was milled for powder X-ray diffraction (XRD), performed using a PANalytical X’Pert Pro instrument, with a Cu*K*α source and a proportional point detector (PW 3011/20). The instrument, housed at the Instituto de Geociências, Universidade Federal de Minas Gerais, was operated at 40 kV and 45 mA. Data collection was in Bragg–Brentano geometry from 5° to 69° (2θ), with 0.02° (2θ) steps at 0.5 s per step. All XRD data are presented in Supplementary Information Table S1 and Fig. S1.

Raman spectra were collected on three representative rutile grains using a Bruker Senterra spectrometer, coupled to an Olympus BX51 light microscope, at the Institute of Applied Geosciences, Karlsruhe Institute of Technology (KIT), Germany. A 532-nm laser, the power of which was 2 mW, with aperture of 50 µm and total time of 30 s (3 s times 10 coadditions), was focused onto specimens with a 20×-microscope lens (OLYMPUS M-PLAN 20x), resulting in a spot diameter of approximately 5 µm on the sample surface.

Measurements for U–Th–Pb isotopes in zircon and rutile were performed in situ on a polished thin section, using a 193-nm ArF Excimer laser (Analyte Exite+, Teledyne Photon Machines), coupled to a Thermo-Scientific Element XR instrument at the KIT. Twenty zircon grains, < 60 µm in length, were large enough for LA–ICP–SF–MS dating using a laser spot size of 20 µm. The grains are rounded and some of them show oscillatory zoning typical of magmatic zircon. Rutile, brownish-red colour in transmitted light (Fig. 3a), was analysed with a laser spot size of 50 µm on grains that were free of optically discernible inclusions and pores (Fig. 3b). Optical microscopy was complemented by scanning electron microscopy (TESCAN VEGA2 SBH with Oxford SwiftED EDS system) for locating sufficiently large, inclusion-free zircon and rutile grains.

Zircon grains of unknown age were dated together with the reference zircon material BB (primary standard), Plešovice and KA (KaapValley), using a laser spot diameter of 20 µm, a laser fluence of 2.7 J/cm^2^, at 10-Hz repetition rate, RF = 1255 W, a mixed Ar–He–N_2_ carrier gas consisting of Ar = 0.965 l/min, He = 0.30 cell + 0.21 cup (both l/min), and N_2_ = 13 ml/min. Three pulses of pre-ablation were performed prior to each analysis of 15-s duration, following 15-s background measurement. All raw data were corrected offline for daily instrumental drift and mass offset employing an in-house MS Excel© spreadsheet programme^34,35^. We applied a common-Pb correction based on the interference- and background-corrected ^204^Pb signal, and a model Pb composition^36^. Results of U–Pb dating of reference zircon and unknows are shown in Supplementary Information Table S2. Multiple measurements of the reference zircon BB (primary standard), Plešovice and KA (KaapValley) (secondary standards) yielded Concordia ages of 561.6 ± 3.2 Ma (MSWD = 0.1, probability = 0.98, n = 10), 338.9 ± 1.4 Ma (MSWD = 0.1, probability = 0.99, n = 10), and 3225.8 ± 7.9 Ma (MSWD = 5.3, probability = 0.02, n = 10), respectively, in agreement with published TIMS ages^37–39^.

Rutile grains of unknown age were dated using the same instrument and setup conditions that are described above. Rutile R10^40^ was employed for matrix-matched primary standard to correct for instrumental ^206^Pb/^238^U mass offset. Multiple analyses of the reference rutile (R10) gave a Concordia age of 1089.8 ± 7.4 Ma (MSWD = 0.21, probability = 0.65, n = 10), in agreement with published TIMS values^40^. Results of U–Th–Pb-isotope analyses of reference rutile and unknowns are shown in Supplementary Information Table S3. Concordia and Tera-Wasserburg diagrams were plotted by means of the software ISOPLOT 4.15^41^. Operation conditions are detailed in Supplementary Information Table S4.

## Supplementary Information

Supplementary Figure S1.

Supplementary Table S1.

Supplementary Table S2.

Supplementary Table S3.

Supplementary Table S4.

## Data Availability

All raw data used for this manuscript are included within the Supplementary Information files.
