# Supplementary material for: Karst-bauxite formation during the Great Oxidation Event indicated by dating of authigenic rutile and its thorium content
Source: Sci Rep. 2023 May 27;13:8633. doi: 10.1038/s41598-023-35574-x (PMC10224920; doi:10.1038/s41598-023-35574-x)
Supplement: Supplementary file 1 — Supplementary Figure S1. [file 41598_2023_35574_MOESM1_ESM.docx]

**Karst-bauxite formation during the Great Oxidation Event indicated by dating of authigenic rutile and its thorium content**

Alexandre Raphael Cabral^1,2^ & Armin Zeh^3*^

^1^ Centro de Pesquisas Professor Manoel Teixeira da Costa (CPMTC), Instituto de Geociências, Universidade Federal de Minas Gerais (UFMG), Belo Horizonte, Brazil

^2^ Centro de Desenvolvimento da Tecnologia Nuclear (CDTN), Belo Horizonte, Brazil

^3^ Karlsruher Institut für Technologie (KIT), Campus Süd, Institut für Angewandte Geowissenschaften, Mineralogie und Petrologie, Karlsruhe, Germany

*Corresponding author: armin.zeh@kit.edu

**Supplementary Information Figure S1**


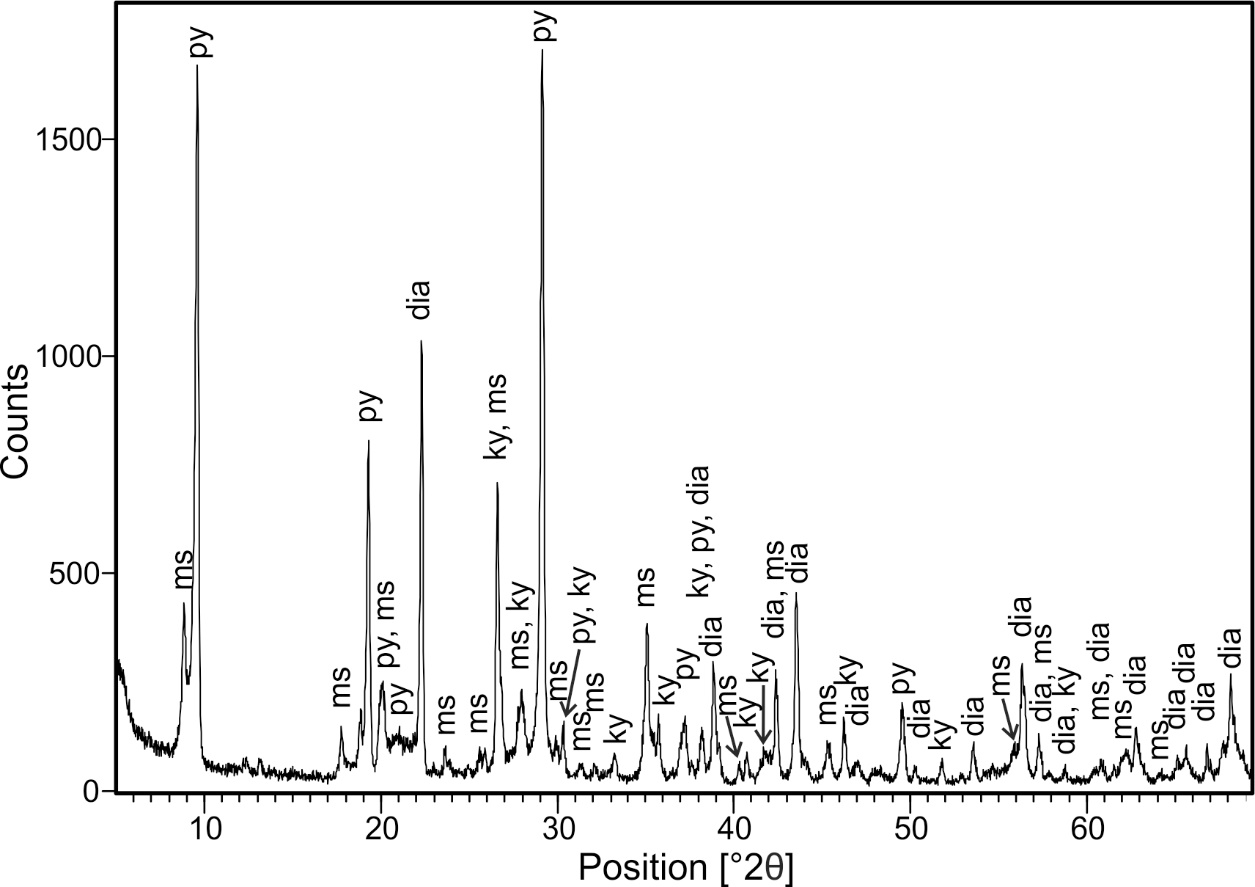


Plot of XRD data from an Al-rich rock (Supplementary Information Table S1). Abbreviations: dia = diaspore; ms = muscovite; ky = kyanite; py = pyrophyllite.
