# Supplementary material for: Karst-bauxite formation during the Great Oxidation Event indicated by dating of authigenic rutile and its thorium content
Source: Sci Rep. 2023 May 27;13:8633. doi: 10.1038/s41598-023-35574-x (PMC10224920; doi:10.1038/s41598-023-35574-x)
Supplement: Supplementary file 5 — Supplementary Table S4. [file 41598_2023_35574_MOESM5_ESM.docx]

**Karst-bauxite formation during the Great Oxidation Event indicated by dating of authigenic rutile and its thorium content**

Alexandre Raphael Cabral^1,2^ & Armin Zeh^3*^

^1^ Centro de Pesquisas Professor Manoel Teixeira da Costa (CPMTC), Instituto de Geociências, Universidade Federal de Minas Gerais (UFMG), Belo Horizonte, Brazil

^2^ Centro de Desenvolvimento da Tecnologia Nuclear (CDTN), Belo Horizonte, Brazil

^3^ Karlsruher Institut für Technologie (KIT), Campus Süd, Institut für Angewandte Geowissenschaften, Mineralogie und Petrologie, Karlsruhe, Germany

*Corresponding author: armin.zeh@kit.edu

**Supplementary Information Table S4. Operating conditions of LA–ICP–SF–MS for U–Th–Pb-isotope analysis**

| **Laboratory** |  |
| --- | --- |
| Laboratory name | Institute for Applied Geosciences, KIT (Karlsruhe Institute of Technology), Germany |
| Imaging | VEGA TESCAN with Oxford detector, BSE mode 15 kV, <10 nA (KIT) |
| **Laser-ablation system** |  |
| Model & type | Teledyne Photon Machines, Analyte Exite+ (Excimer) |
| Ablation cell | Two-volume ablation cell (HELEX 100, EQC COMP), ANUAustralia) |
| Laser wavelength | 193 nm |
| Pulse width | <5 ns |
| Fluence | 2.7 J/cm^-2^ |
| Repetition rate | 8 Hz |
| Spot size | 40 µm |
| Sampling mode / pattern | single spot |
| Carrier gas | He (cell) 0.30 l/min, He (cup) 0.2 l/min, 0.978 l/min Ar, N_2_ (12 ml) |
| Background collection | 15 seconds |
| Ablation duration | 15 seconds |
| Pre-ablation | 3 pulses |
| Wash-out delay | 25 seconds |
| Cell carrier gas flow (He) | 0.50 l/min (total) |
| **ICP–MS Instrument** |  |
| Model & type | Thermo-Scientific ELEMENT XR (sector field) |
| Sample introduction | via conventional tubing |
| RF power | 1240 W |
| Sampler, skimmer cones | Ni-X, Jet cone |
| Extraction lenses | X type |
| Make-up gas flow (Ar) | 0.98 l/min |
| Detection system | single collector secondary electron multiplier |
| Data acquisition protocol | Time-resolved analysis |
| Scanning mode | Peak hopping, four point per peak |
| Detector mode | Pulse counting mode |
| Masses measured | ^202^Hg,^204^(Hg + Pb), ^206^Pb, ^207^Pb, ^208^Pb, ^232^Th, ^238^U |
| Integration time per peak | 10 milliseconds |
| Sensitivity / Efficiency | 22500 (cts/ppm) (^238^U, NIST612, spot-35μm, 10μm/s, 10 Hz, 5 J/cm^2^)  oxide formation rate: U/UO < 0.08, Th/U = 0.99 |

| **Data Processing** |  |
| --- | --- |
| Gas blank | 15 seconds on peak |
| Calibration strategy | BB zircon standard used as primary reference material, Plešovice, KA (Kaap Valley tonalite) as secondary zircon reference material  R10 rutile was used as primary standard for rutile dating |
| Reference Material info | BB (Santos et al., 2017): concordia age = 562.0 ± 0.6 Ma (LA-ICP-MS)  Plešovice (Slama et al., 2008) = 337.13 ± 0.37 Ma (TIMS)  KA1 (Schoene et al. (2006): 207/206 = 3227.2 ± 0.2 Ma (CA-ID-TIMS).  R10 (Luvizotto et al., 2009): ^207^Pb/^235^U = 1085.1 to 1096.2 Ma,  ^206^Pb/^238^U = 1086.3 to 1096.6 Ma (TIMS) |
| Data processing package used | in-house EXCEL spreadsheet (Gerdes and Zeh, 2006, 2009) |
| Quality control / Validation | BB: concordia age = 561.6 ± 3.2 Ma (n = 10)  Plešovice: concordia age = 338.9 ± 2.1 Ma (n = 10)  KA1: 207/206 age = 3225.8 ± 7.9 Ma (n = 10)  R10: concordia age = 1089.8 ± 7.4 Ma (n = 10) |
